# Characterization of HIV-1 subtypes in a Portuguese cohort

**DOI:** 10.7448/IAS.17.4.19683

**Published:** 2014-11-02

**Authors:** Nuno Rocha Pereira, Raquel Duro, Carmela Piñero, Luís Malheiro, Jorge Soares, Rosário Serrão, António Sarmento

**Affiliations:** Infectious Diseases, Centro Hospitalar São João, Porto, Portugal

## Abstract

**Introduction:**

Distribution of HIV-1 subtypes is variable around the world, with the most common subtype in western Europe being subtype B. The aim our study was to describe the prevalence of different HIV-1 subtypes in newly diagnosed patients and identify demographic and epidemiological characteristics related with different subtypes.

**Materials and Methods:**

Retrospective single-centre study of patients newly diagnosed with HIV-1 infection between 2006 and 2012. Epidemiological data was gathered and genotyping was performed in each patient identified. Demographic and epidemiological characteristics were compared between patients with subtype B and other subtypes. Continuous variables were summarized by mean and standard deviation whereas categorical variables were presented as proportions. Comparison of groups was performed using the Chi square, Fisher exact test and Student T test. Statistical significance was assumed when p<0.05.

**Results:**

In the period of the study, 624 patients newly diagnosed with HIV-1 infection were submitted to genotypic testing but information about subtype was available only for 592 patients. General characteristics of the patients are summarized in Table 1. The distribution of the identified subtypes was the following: 286 (48.3%) patients had subtype B, 157 (26.5%) had subtype G, 54 (9.1%) had subtype C, 36 (6.1%) had subtype A, 32 (5.4%) had subtype F and 25 (4.2%) had CRF's. Patients with subtype B were more commonly male (p=0.001) and younger (p<0.0001) than those with subtypes other than B. Subtype B was more common in MSM patients, while non-B subtypes were more common in heterosexual patients and in injecting drug users (p=0.001). CD4-cell count, viral load and AIDS at presentation were not significantly different between subtypes. Resistance associated mutations were significantly more common in patients with non-B subtypes (15.4% vs 9.8%; p=0.048).

**Conclusions:**

The most commonly identified subtype was B in accordance with previous reports from other western European countries. However, in our cohort the proportion of non-B subtypes is higher than that reported for other European countries, probably reflecting the influence of strong bonds with Portuguese speaking African countries. Knowledge about HIV subtypes distribution may help understanding transmission dynamics and can be an important tool in the design of preventive measures.

**Table 1:** General characteristics of the patients

Characteristics	n=592

Sex	
Male	425 (71.8%)
Female	167 (28.2%)
Age (mean±SD)	40.78±13.45
Risk for HIV acquisition	
Heterosexual	410 (69.5%)
MSM	132 (22.2%)
Injecting drug user	46 (7.8%)
Others	2 (0.3%)
CD4-cell count /mL (mean±SD)	320±257
HIV-1 Viral load copies/mL (mean±SD)	7,23,035±19,70,266

MSM, Men that have sex with men; SD, Standard deviation; CD4, CD4+ T cell lymphocytes.

